# Insight into the Draft Genome Sequence of Human Isolate *Lactobacillus rhamnosus* LR231, a Bacterium with Probiotic Potential

**DOI:** 10.1128/genomeA.00111-14

**Published:** 2014-02-27

**Authors:** Padma Ambalam, Sheetal Pithva, Charmy Kothari, Ramesh Kothari, Nidhi Parmar, Neelam M. Nathani, Prakash G. Koringa, Chaitanya G. Joshi, J. M. Dave, B. R. M. Vyas

**Affiliations:** a Department of Biotechnology, Christ College, Gujarat, India; b Department of Biosciences, Saurashtra University, Rajkot, Gujarat, India; c Department of Microbiology, Christ College, Gujarat, India; d Department of Animal Biotechnology, Anand Agricultural University, Anand, Gujarat, India; e Vrindavan Society, Rajkot, India

## Abstract

*Lactobacillus rhamnosus* strain LR231 was isolated from the feces of healthy human subjects. It is observed to be a potential probiotic strain, having a broad spectrum of antimicrobial activity against a wide range of human pathogens and food pathogens. Here, we provide the 2.59-Mb draft genome sequence of *L. rhamnosus* LR231.

## GENOME ANNOUNCEMENT

The human body constitutes a huge consortium of microbes, the majority of them inhabiting the lower part of the gastrointestinal (GI) tract. Hence, humans are programmed by their own genome as well as by the environmentally acquired microbiome (1). Various functions of intestinal microbes impact our lives, like food metabolism, antagonist effect on pathogens, and several signaling functions (2, 3). Hence, the intestinal microbiota has a significant influence on various aspects of the host physiology and metabolism, and it is also possible to modulate the genetic composition by changing the environmental conditions, which ultimately leads to a change in the microbial diversity and functions (4).

The consumption of lactic acid bacteria marketed as probiotics is a common approach to maintaining health (5). Currently, probiotics are defined as microorganisms that confer a health benefit on the host when administered in adequate amounts (6). Various minimum criteria have been defined for a strain to be designated a probiotic, such as the microorganism being generally recognized as safe (GRAS), being able to survive the low pH of the GI tract and to adhere to human intestinal cells, being antagonistic to potential pathogens, and having beneficial effects to host (7).

*Lactobacillus rhamnosus* LR231 is a potential probiotic strain, having a broad spectrum of antimicrobial activity against a wide range of human pathogens and food pathogens. Potential probiotic human strain *L. rhamnosus* LR231 was shown to possess antimicrobial activity against several human pathogens (8).

Whole-genome shotgun sequencing was performed using the 318 Chip and 300-bp chemistry Ion Torrent PGM platform as per the manufacturer’s instructions. The draft genome of *L. rhamnosus* LR231 showed the presence of 183 contigs of >200 bp in size when the obtained sequence reads were subjected to reference-guided assembly against the whole-genome sequence of the reference organism *L. rhamnosus* GG using GS Reference Mapper software version 2.3.

The gene annotation and screening for RNAs were performed by submitting the sequences to the Rapid Annotations using Subsystems Technology (RAST) server (9). Consequently, 2,607 protein-coding sequences (CDSs) were identified, of which 2,290 CDSs were assigned to one of the 291 RAST subsystems. The genome contains 67 RNA molecules.

The genome analysis showed the strain to possess a relatively high number of CDSs involved in carbohydrate and amino acid metabolism, transport, and virulence-defense mechanisms. The genome contains 40 CDSs encoding about 13 complete phosphoenolpyruvate-carbohydrate phosphotransferase-type transporter systems (PTSs). The organism carries 66 coding sequences related to proteins and enzymes involved in ABC transporters, 7 CDSs coding for antibacterial peptides, and 6 β-lactamases, which thus confirm its broad range of antimicrobial activity and antibiotic resistance properties. Further, this strain exhibits *in vitro* binding of *N*-methyl-*N*′-nitro-*N*-nitrosoguanidine (MNNG) and 2-amino-3,8-dimethylimidazo[4,5-f]quinoxaline (MeIQx) and biotransformation, as well as subsequent detoxification and antimutagenic activity (10). Administration of viable LR231 protected rats from MNNG-induced colon inflammation (11). The safety of this strain has been proven in a mouse model (12). Thus, the information obtained from the genome sequence about the various genes involved in the functioning of various metabolic pathways and defense mechanisms will give a better understanding of the antimicrobial activities and probiotic potentials of the strain.

### Nucleotide sequence accession number.

The sequence of *L. rhamnosus* LR231 has been deposited at GenBank under the accession no. AZHJ00000000.
